# Genome-wide association studies (GWAS) identify a QTL close to *PRKAG3* affecting meat pH and colour in crossbred commercial pigs

**DOI:** 10.1186/s12863-015-0192-1

**Published:** 2015-04-07

**Authors:** Chunyan Zhang, Zhiquan Wang, Heather Bruce, Robert Alan Kemp, Patrick Charagu, Younes Miar, Tianfu Yang, Graham Plastow

**Affiliations:** Department of Agricultural, Food & Nutritional Sciences, University of Alberta, Edmonton, AB T6G 2P5 Canada; Genesus Inc, Oakville, MB Canada; Hypor Inc, Regina, SK Canada

**Keywords:** Genome-wide association studies, Meat pH and colour, Pig, SNP

## Abstract

**Background:**

Improving meat quality is a high priority for the pork industry to satisfy consumers’ preferences. GWAS have become a state-of-the-art approach to genetically improve economically important traits. However, GWAS focused on pork quality are still relatively rare.

**Results:**

Six genomic regions were shown to affect loin pH and Minolta colour a* and b* on both loin and ham through GWAS in 1943 crossbred commercial pigs. Five of them, located on *Sus scrofa* chromosome (SSC) 1, SSC5, SSC9, SSC16 and SSCX, were associated with meat colour. However, the most promising region was detected on SSC15 spanning 133–134 Mb which explained 3.51% - 17.06% of genetic variance for five measurements of pH and colour. Three SNPs (ASGA0070625, MARC0083357 and MARC0039273) in very strong LD were considered most likely to account for the effects in this region. ASGA0070625 is located in intron 2 of *ZNF142,* and the other two markers are close to *PRKAG3, STK36, TTLL7* and *CDK5R2.* After fitting MARC0083357 (the closest SNP to *PRKAG3*) as a fixed factor, six SNPs still remained significant for at least one trait. Four of them are intragenic with *ARPC2, TMBIM1, NRAMP1* and *VIL1*, while the remaining two are close to *RUFY4* and *CDK5R2.* The gene network constructed demonstrated strong connections of these genes with two major hubs of *PRKAG3* and *UBC* in the super-pathways of cell-to-cell signaling and interaction, cellular function and maintenance. All these pathways play important roles in maintaining the integral architecture and functionality of muscle cells facing the dramatic changes that occur after exsanguination, which is in agreement with the GWAS results found in this study.

**Conclusions:**

There may be other markers and/or genes in this region besides *PRKAG3* that have an important effect on pH and colour. The potential markers and their interactions with *PRKAG3* require further investigation

**Electronic supplementary material:**

The online version of this article (doi:10.1186/s12863-015-0192-1) contains supplementary material, which is available to authorized users.

## Background

Improving meat quality has become a high priority for the pork industry to satisfy consumers’ preferences for a better eating experience. This is vital for the swine industry to sustain profitability and enhance its competiveness in terms of both swine genetics and sales of pork (including export markets). Minolta colour and pH are two common industry measures of pork quality that are directly correlated with glycogen and glycolytic potential [1]. Colour and pH are also highly correlated with other pork quality measurements (e.g. drip loss, texture score) and carcass yield (e.g. carcass weight, loin depth, loin length) as reported in our recent work [2]. However, improving these traits by traditional breeding methods is a challenge due to the wide range of reported heritability (0.04 - 0.57) in different breeds [3], the high cost of measurement and the limited amount of data available post-mortem. In recent decades, nearly 900 QTLs (PigQTLdb, release 25, December 2014. http://www.animalgenome.org/cgi-bin/QTLdb/SS/index) and a few gene variants, such as *PRKAG3* (Protein Kinase, AMP-Activated, Gamma 3 Non-Catalytic Subunit) [1,4,5], have been found to affect pork pH and colour, which provides a new breeding strategy to improve these traits on the basis of DNA markers.

Most of the QTLs identified were detected using linkage mapping and cover large regions of the genome. With the availability of high-density SNP chips and genome wide analysis methodology, GWAS have been increasingly used to identify more precisely the genomic regions and markers associated with quantitative traits, and have begun to be used in dairy cattle and poultry breeding programs as reviewed by Goddard and Hayes [6]. Recently, additional GWAS have been reported in pigs and several genomic regions have been identified for different traits [7-13]. However, because of the difficulty and expense of measurement for meat quality, GWAS focused on these traits have still been limited for now [14-18].

Therefore, the aims of this study were to detect genomic regions and identify potential candidate genes and markers affecting pork pH and colour by combining GWAS and post-GWAS bioinformatics analysis. The results will contribute useful information for marker based genomic improvement of these traits. Knowledge of the gene networks will provide new insights for the biological mechanisms underlying these traits.

## Results

### Genomic regions

Population stratification with Identity-by-State (IBS) showed that all animals from the two commercial populations could be classified into two clusters, which correspond to the populations from which the individuals were derived. Therefore, population was fitted as a fixed effect to optimize the association analysis model.

Genomic regions affecting both fresh and thawed loin pH, colour a* and b* on fresh ham, and colour b* on thawed loin muscle were detected by Bayes B 1 Mb window analysis. Accordingly, a larger proportion of genetic variance explained by the region indicates a stronger effect of the corresponding region on the target trait. The genomic regions that explained more than 1% of the total genetic variance for the traits studied are shown in Table 1. The most promising genomic region associated with the pH and colour was located on SSC15 between 133 - 134 Mb (15_133). A total of 26 SNPs in this region explained the major proportion of the total genetic variance for the five traits, with 17.06%, 8.68%, 12.91%, 9.26% and 3.51% for colour b* (QFCOL b*) and a* (QFCOL a*) measured on the *quadriceps femoris* on the ham, colour b* measured on the thawed loin (TMCOL b*), pH 24 h post-mortem measured on fresh loin (FpH24) and pH measured on thawed loin (TMpH), respectively. Other genomic regions on SSC1, SSC5, SSC9, SSC16 and SSCX also explained a relatively large proportion of genetic variance of colour. For example, the region on SSC5 (5_107) explained 4.44% of the genetic variance for QFCOL a*, and each of the others explained about 1% of the total genetic variance for QFCOL a* and TMCOL b*.

**Table 1:** **Promising chromosome regions for pH and colour detected by Bayes B analysis**

**Traits**	**Regions (SSC and position (Mb))**	**1** ^**st**^ **SNP**	**Last SNP**	**No. of SNPs**	**% Genetic var.** ^**#**^
QFCOL b*	15_133	ASGA0070560	ASGA0070646	26	17.06
	9_147	MARC0054470	H3GA0028692	30	1.36
QFCOL a*	15_133	ASGA0070560	ASGA0070646	26	8.68
	5_107	MARC0078506	H3GA0017340	25	4.44
	1_35	H3GA0001359	MARC0036030	25	1.25
TMCOL b*	15_133	ASGA0070560	ASGA0070646	26	12.91
	16_73	ASGA0073971	M1GA0021138	25	1.25
	X_1	ASGA0083984	ALGA0098972	19	1.00
FpH24	15_133	ASGA0070560	ASGA0070646	26	9.26
TMpH	15_133	ASGA0070560	ASGA0070646	26	3.51

^#^percentage of the genetic variance explained by each region.

*indicates the traits of Minolta colour a* and Minolta colour b*, which are the standard description for these traits (a*, b*).

### Detailed analysis of the region on SSC15

**Figure 1:**
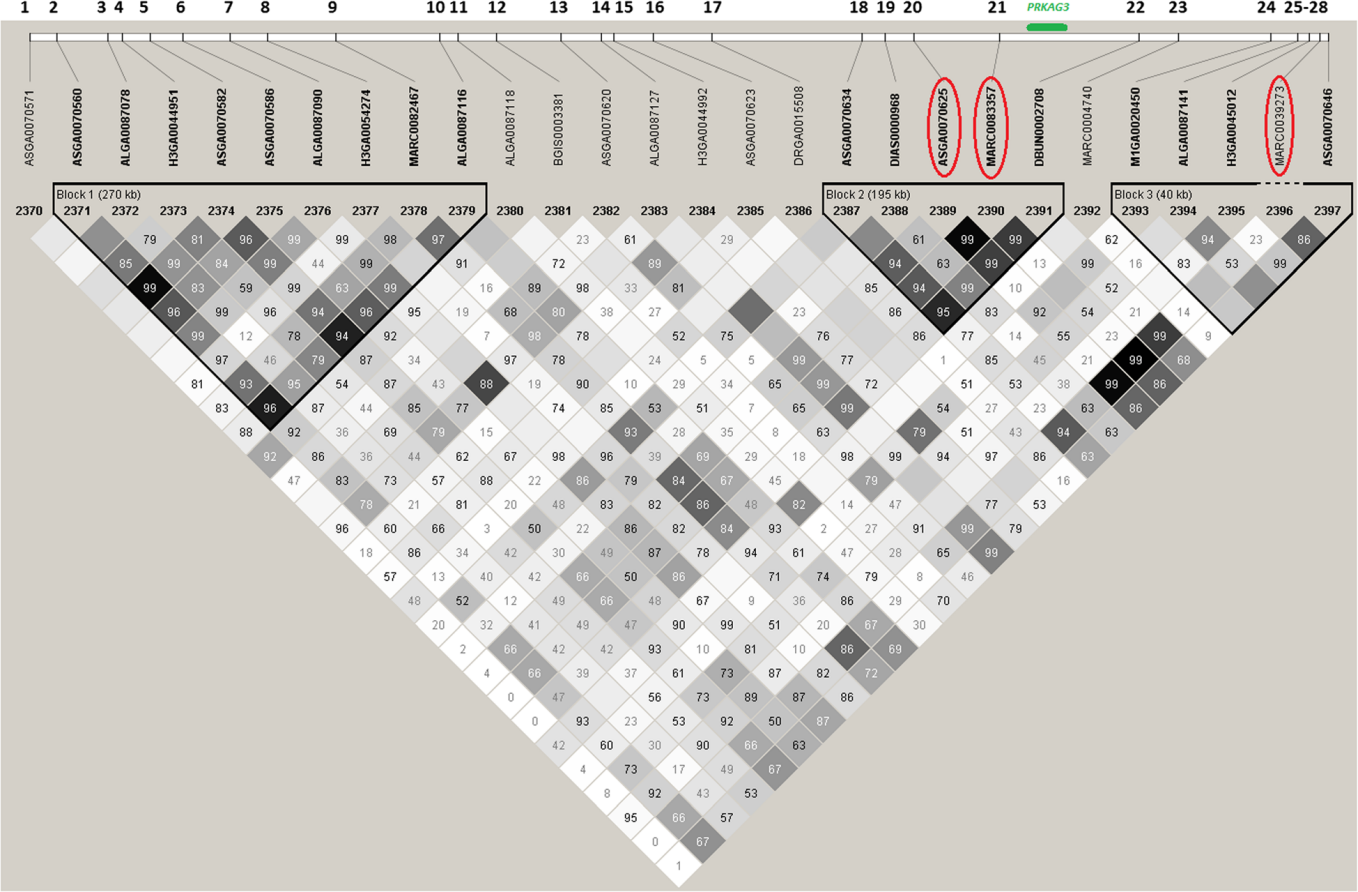
**Haplotype block pattern (r** ^**2**^ **-scheme) for the region SSC15_133 based on the LD (r** ^**2**^ **) among the 28 SNPs within this region.** The numbers on the top indicate the SNP order in this region; SNPs in the red circles are the most likely markers detected from GWAS; The green line indicates the approximate map position of the *PRKAG3* gene; The SNPs grouped in each triangle box means they are grouped in one block based on LD information.

The region of SSC15_133 spans 898,069 base pairs and includes a total of 28 SNPs on the 60K SNP panel. Two SNPs (1 and 17) with low genotype call rates were filtered out from the analysis. An overview of the distribution of all the SNPs and their relationships is shown in the LD haplotype map in Figure 1. Out of the three haplotype blocks identified, block 2 consisting of five SNPs (18 - 22) is the most interesting one as it harbors *PRKAG3* which was previously reported as a candidate gene associated with meat quality. The LD between block 2 and SNP27 is also strong.

For further study, window (15_133) GEBVs for the traits were summed based on the marker effects of the 26 SNPs, and then regressed on the genotype covariate for each SNP (0/1/2). Three SNPs (20, 21 and 27) with almost complete LD captured an average of 98.2%, 99.2% and 99.0% variances of window GEBVs. These results indicated that any of the three SNPs could be affecting the traits. To further analyze these markers, SNP21 (MARC0083357) was chosen as a fixed effect in the animal model (ASREML) to estimate its allele substitution effect. The results (Table 2) showed that SNP21 had a significant effect (P < 0.0001) on all traits. Allele B has positive effects on QFCOL a*, QFCOL b* and TMCOL b*, and negative effects on both FpH24 and TMpH. Further analysis indicated that SNP21 can capture almost all of the phenotypic variance that is explained by this region, e.g. 1% for pH and 1.38% - 1.75% for colour. While, as shown in Table 2, the percentage of phenotypic variance explained by SNP21 (from ASREML) is even slightly higher than that explained by the region. This is because the proportions of phenotypic variance explained by the window were calculated based on the molecular heritability (the percentage of the whole phenotypic variance explained by the fitted SNPs). It is usually considered that the molecular heritability based on the genotypes is smaller than that estimated based on pedigree due to the missing values from the unconsidered markers.

**Table 2:** **Effects and proportions of phenotypic variance explained by SNP21 (MARC0083357)**

**Trait**	**Effect (B allele)**	**Standard error**	**% Phenotypic var. explained by SNP21** ^**#1**^	**% Phenotypic var. explained by the region of SSC15_133** ^**#2**^
QFCOL b*	0.249	0.042	1.65	1.54
QFCOL a*	0.248	0.048	1.38	1.13
TMCOL b*	0.181	0.030	1.75	1.68
FpH24	−0.018	0.003	1.01	1.30
TMpH	−0.010	0.003	0.52	0.35

^#1^calculated on the basis of ASREML results, as 2pqB^2^ of SNP21 (MAF = 0.50) divided by the total phenotypic variance; ^#2^calculated on the basis of Bayes B results, as the molecular heritability multiplied by the percentage of the whole genetic variance explained by the window as shown in Table 1.

*indicates the traits of Minolta colour a* and Minolta colour b*, which are the standard description for these traits (a*, b*).

To investigate other SNPs in this region, each SNP was fitted as a fixed effect to test their statistical significance on the traits after the effect of SNP21 was accounted for in the model. The *P_values* for the significance test of all the SNPs are shown in Table 3. Only a few of the SNPs remained significant (P < 0.05) after accounting for the effect of SNP21, such as SNP12 for QFCOL b*; SNPs 9, 13, 19 and 28 for QFCOL a*; SNPs 16 and 18 for TMCOL b*; SNPs 14, 18, 19, 22, 23 and 24 for TMpH. To reduce the overestimation of single marker regression, all these significant SNPs, including SNP21, were fitted as fixed effects in the model simultaneously to retest their significance. The results showed that a reduced number of SNPs remained significant (P < 0.05). These were SNPs 12 and 21 for QFCOL b*; SNPs 9, 13 and 21 for QFCOL a*; SNPs 16 and 21 for TMCOL b*; SNPs 14, 21 and 24 for TMpH.

**Table 3:** **Single marker statistical significance test (** ***P_values*** **) for other SNPs in the SSC15_133 region after accounting for the effect of SNP21 (MARC0083357)**

**SNP_ID**	**SNP_name**	**Position**	**MAF**	**LD with SNP21**	***P_values***				
					QFCOL b*	QFCOL a*	TMCOL b*	**FpH24**	**TMpH**
2	ASGA0070560	133072097	0.44	0.36	0.775	0.971	0.896	0.755	0.560
3	ALGA0087078	133108407	0.36	0.12	0.905	0.739	0.234	0.796	0.965
4	H3GA0044951	133118381	0.36	0.15	0.757	0.616	0.797	0.652	0.587
5	ASGA0070582	133138277	0.43	0.35	0.794	0.979	0.994	0.682	0.875
6	ASGA0070586	133160977	0.48	0.24	0.860	0.987	0.916	0.967	0.441
7	ALGA0087090	133194513	0.28	0.30	0.473	0.860	0.949	0.586	0.215
8	H3GA0054274	133220838	0.18	0.14	0.189	0.573	0.821	0.808	0.991
9	MARC0082467	133269167	0.44	0.63	0.411	**0.003**	0.090	0.458	0.170
10	ALGA0087116	133342361	0.43	0.35	0.922	0.900	0.432	0.786	0.614
11	ALGA0087118	133355327	0.15	0.01	0.348	0.488	0.484	0.840	0.157
12	BGIS0003381	133382636	0.20	0.00	**0.004**	0.239	0.956	0.090	0.889
13	ASGA0070620	133427999	0.31	0.21	0.520	**0.034**	0.460	0.530	0.428
14	ALGA0087127	133456604	0.30	0.41	0.908	0.137	0.966	0.555	**0.010**
15	H3GA0044992	133465593	0.18	0.14	0.077	0.449	0.619	0.847	0.847
16	ASGA0070623	133493709	0.15	0.21	0.953	0.373	**0.020**	0.195	0.606
18	ASGA0070634	133640599	0.42	0.69	0.999	0.135	**0.050**	0.656	**0.003**
19	DIAS0000968	133656928	0.38	0.28	0.848	**0.014**	0.455	0.162	**0.002**
20	ASGA0070625	133677385	0.49	0.97	N/A	N/A	N/A	N/A	N/A
21	MARC0083357	133738342	0.50	Fixed	N/A	N/A	N/A	N/A	N/A
22	DBUN0002708	133836471	0.44	0.78	0.911	0.078	0.060	0.442	**0.008**
23	MARC0004740	133864581	0.10	0.00	0.307	0.103	0.597	0.723	**0.013**
24	M1GA0020450	133929898	0.20	0.27	0.211	0.858	0.602	0.484	**0.047**
25	ALGA0087141	133948641	0.34	0.16	0.909	0.220	0.080	0.969	0.179
26	H3GA0045012	133956838	0.21	0.02	0.997	0.154	0.076	0.855	0.128
27	MARC0039273	133964455	0.50	1.00	N/A	N/A	N/A	N/A	N/A
28	ASGA0070646	133970166	0.46	0.63	0.110	**0.020**	0.576	0.455	0.961

Note: bold data means P < 0.05.

*indicates the trait name of Minolta colour a* and b*.

**Figure 2:**
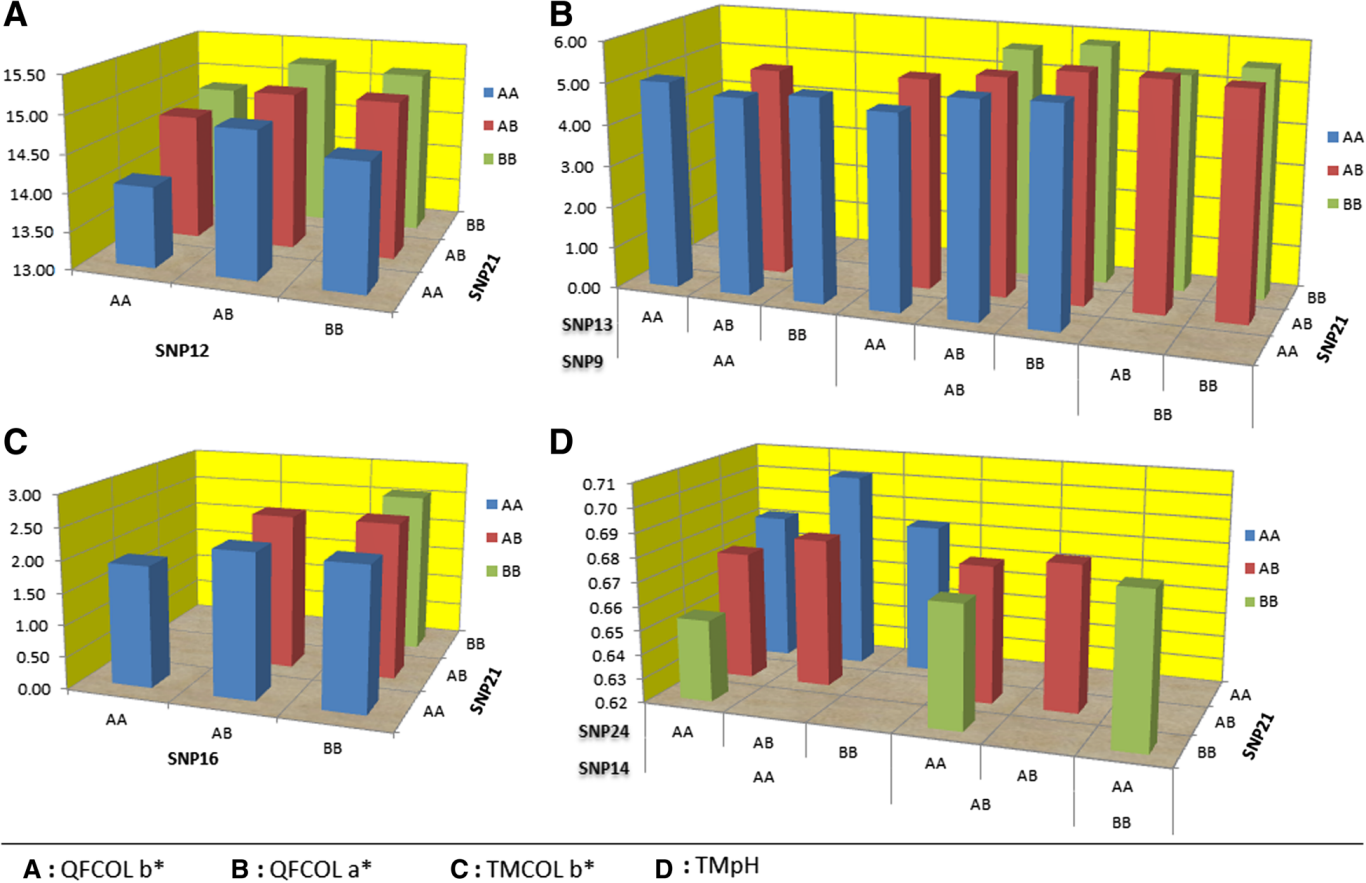
**Least squares mean (LSMs) for the haplotypes constructed for the significant SNPs from multiple-marker association. (A)**: LSMs of QFCOL b* for the haplotypes constructed by SNP21 and SNP12. AA, AB and BB are the three genotypes for each SNP. **(B)**: LSMs of QFCOL a* for the haplotypes constructed by SNP21, SNP13 and SNP9. **(C)**: LSMs of TMCOL b* for the haplotypes constructed by SNP21 and SNP16. **(D)**: LSMs of TMpH for the haplotypes constructed by SNP21, SNP24 and SNP14.

Haplotypes were constructed for the significant SNPs identified in the multiple marker association analysis. For FpH24, none of the other SNPs was significant after fitting SNP21 in the model. The least squares means (LSMs) of the haplotypes for other traits are shown in Figure 2. For QFCOL b* (Figure 2 (A)), the B allele of SNP21 had a positive effect (BB > AB > AA). SNP12 showed an over-dominance effect. Therefore, the genotype BBAB (SNP 21–12) had the largest value for colour b* and AAAA animals the lowest value.

For QFCOL a* (Figure 2 (B)), three SNPs (21, 9 and 13) remained significant and resulted in 16 major haplotypes accounting for 99% of the whole sample. For the haplotype SNP 21-9-13, the largest value was observed for BBABBB animals (n = 19) and the lowest value was for the animals with AAABAA (n = 23) or AAAAAB (n = 208). However, the small sample size for these groups might have resulted in a larger standard error. These results need further validation in a larger sample size.

For TMCOL b* (Figure 2 (C)), SNP21 and SNP16 remained significant and resulted in 6 haplotypes. Individuals with BBBB (n = 509) accounted for 26.2% of the population and had the highest b* value. Individuals with AAAA genotype (n = 50) showed the lowest value. However further study is encouraged due to the small number of AAAA animals in this sample.

For TMpH (Figure 2 (D)), three SNPs of 21, 14 and 24 remained significant and resulted in 10 haplotypes. Haplotype AAAAAB (SNP 21-14-24) (n = 257) had the highest pH compared with the haplotype BBAAAA (n = 87) with the lowest value. However, no significant difference was detected between other haplotypes, which indicated that the main effect seems to be due to SNP21.

### Candidate genes and their functional network

A total of 29 genes including 17 of unknown function were identified in a 1 Mb region on SSC15. The nine significant SNPs (including 20, 21 and 27) were found to be intragenic or close to ten genes as shown in Table 4. SNP21 is close to *ZNF142* and *STK36*, and about 62 kb upstream of *PRKAG3*. For SNP20 and SNP27, which have very high LD with SNP21, one is located in the intron of *ZNF142* (SNP20) and the other is close to *CDK5R2* (SNP27). For the other six SNPs, four are intragenic with *ARPC2, TMBIM1, NRAMP1* and *VIL1*, and two are close to *RUFY4* and *CDK5R2*.

**Table 4:** **The candidate or nearest genes to the significant SNPs in the region on SSC15**

**SNP_ID**	**SNP position**	**Ensembl gene ID**	**Gene start**	**Gene end**	**Gene name**	**Distance (bp)**
9	133269167	ENSSSCG00000016182	133249206	133268676	*RUFY4*	492
12	133382636	ENSSSCG00000016185	133377438	133389903	*ARPC2*	Exon 3
13	133427999	ENSSSCG00000016186	133425398	133432043	*TMBIM1* ^*#*^	Intron 9
14	133456604	ENSSSCG00000025058	133452329	133456736	*NRAMP1*	Exon 4
16	133493709	ENSSSCG00000016196	133479159	133507423	*VIL1*	Intron 10
20	133677385	ENSSSCG00000016191	133667702	133729988	*ZNF142* ^*#*^	Intron 2
21	133738342	ENSSSCG00000016191	133667702	133729988	*ZNF142* ^*#*^	8,354
		ENSSSCG00000026964	133753315	133852318	*STK36* ^*#*^	−14,973
		ENSSSCG00000016201	133768369	133815768	*TTLL7* ^*#*^	−30,027
		ENSSSCG00000016200	133800248	133807019	*PRKAG3*	−61,906
24	133929898	ENSSSCG00000021584	133925849	133927094	*CDK5R2*	2,804
27	133964455	ENSSSCG00000021584	133925849	133927094	*CDK5R2*	37,361

^*#*^unknown genes in *Sus scrofa*, the names in the table are their orthologues in other species.

**Figure 3:**
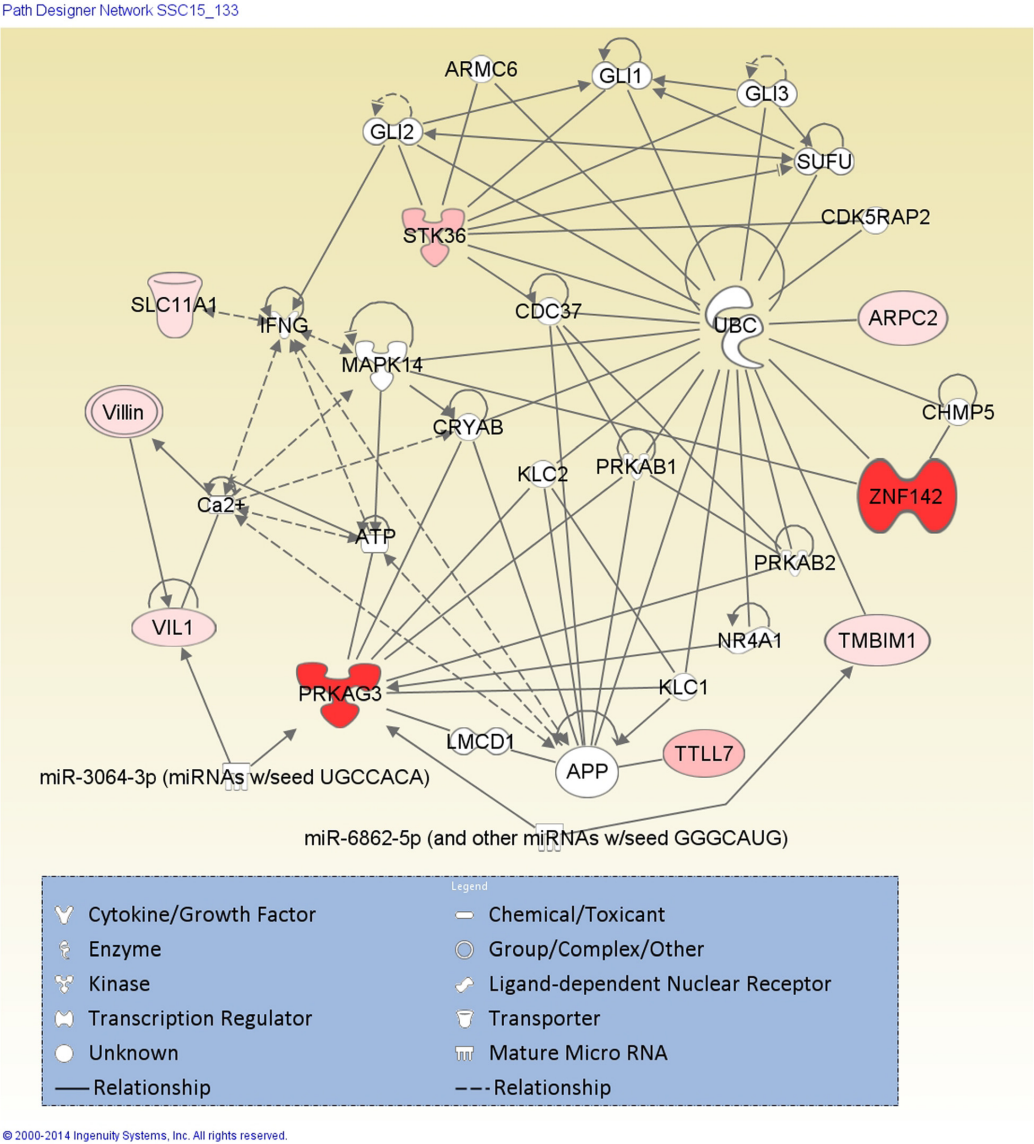
**Gene network constructed based on the candidate and/or nearest genes to the significant SNPs in the SSC15_133 region using IPA software.** The genes marked by red and pink means they are candidate genes or the closest ones to the significant SNPs detected in the region. The darker means the greater contribution involved in the functional pathway. The white ones were selected automatically by IPA procedure based on their functional annotations that are related to the pathways.

A functional network (Figure 3) among these ten genes was constructed based on Gene Ontology (GO) and their functional pathways. *NRAMP1* was replaced by the human orthologues of *SLC11A1* during the process. Except for *RUFY4*, all the genes (coloured by pink and red) were strongly connected through the functional pathways which are involved in cell-to-cell signaling and interaction, cellular function and maintenance. Usually the genes with darker colour indicate they have more contributions to the pathway and have stronger connection to the network. For example, *ZNF142* and *PRKAG3* were coloured dark red indicating the greatest contribution to these pathways compared to the other genes that were coloured light red and pink. Gene annotation (Additional file 1: Table S1) indicated that most of these genes are related to the cellular component of cell-substrate junction, membrane and extracellular region, which play important roles in maintaining the internal architecture of muscle cells through signal transduction and a variety of biological processes. These gene products might play important roles in regulating post-mortem effects that impact muscle pH and colour.

### Analysis of other genomic regions

Candidate genes in the regions identified on other chromosomes are presented in Table 5. Thirteen genes were identified in the region on SSC1 impacting QFCOL a*, where the most likely marker was H3GA0001381 which explained 91.5% of the window GEBV variance. For SSC5, only one known gene (*SYT1)* was detected in this region where MARC0017490 was the most promising marker (RSQ = 0.975) for QFCOL a*. Three genes were found for SSC9_147 where three SNPs with high LD (r^2^ = 0.63 - 0.89) contributed the major effect (each of them explained more than 87% variance of the window GEBV). Only one gene was identified for the region on SSC16 and the most promising SNP was ALGA0091417. For the region on SSCX, three genes were identified and ASGA0083984 was the best marker explaining almost all the variance of window GEBV (RSQ = 0.998).

**Table 5:** **Genes detected in other regions and the most likely SNPs in the regions**

**Regions**	**Most promising SNP**	**Position**	**RSQ** ^**#3**^	**Candidate genes in the region**
1_35Mb	H3GA0001381	35702627	0.915	13 genes with 8 unknown ones: *CTGF, ENPP1, ENPP3, ARG1, 7SK*
5_107Mb	MARC0017490	107260629^**#1**^	0.975	1 gene: *SYT1*
	ALGA0034179	107073577^**#1**^	0.869	
9_147Mb	ALGA0102900	147079835^**#2**^	0.927	3 genes with 2 unknown ones: *U6*
	ALGA0101542	147108528^**#2**^	0.906	
	ASGA0045220	147280710^**#2**^	0.869	
16_73Mb	ALGA0091417	73416075	0.841	1 gene: *5S rRNA*
X_1Mb	ASGA0083984	1141455	0.998	3 genes with 1 unknown one: *OBP, CH242-123G14.1*

^#1^The LD (r2) between the two SNPs is 0.75; ^#2^The LD (r2) between the SNPs ranges from 0.63 to 0.89; ^#3^the R-squared, means the percentage of the window GEBV variance explained by the single SNP.

## Discussion

### Genomic regions for pork pH and colour

In this study, a 1 Mb region (133 – 134 Mb) on SSC15 explained the majority of the genetic variance for loin pH, loin and ham colour a* and b*. Previous linkage mapping studies have reported that SSC15 is enriched for QTLs affecting meat pH and colour. These include five QTLs between 56 cM to 119 cM for ham and loin pH 24 h post-mortem [1,19], and a large region of 88.5 - 122 cM [20] as well as peaks located at 42.21 cM and 44.80 cM [21] for colour b*. A recent GWAS study in the Finnish Yorkshire breed detected a relatively small region (133.64 - 134.01 Mb) on SSC15 associated with meat quality [18]. This region was also identified in this study. These results provide further support for the region of 133 - 134 Mb on SSC15 being a true QTL affecting meat pH and colour, especially as it is found in different populations. A positional candidate gene approach also showed that *PRKAG3*, located within this region, can be considered as one of the few major genes known to affect meat pH and colour. Four missense substitutions (T30N, G52S, I199V and R200Q) in this gene were found to significantly affect meat quality [1,4]. However, since none of these mutations are included in the 60K SNP panel, the contribution of *PRKAG3* to the effect of the region on the traits remains unknown. Therefore, it is essential to extend the analysis of the regions flanking *PRKAG3* and investigate the genomic architecture of this region affecting meat pH and colour.

Five other regions, on SSC1, SSC5, SSC9, SSC16 and SSCX, were also found to capture a relatively large proportion of genetic variance for meat colour in this study, indicating their important effects on these traits. However, the limited gene and map information in these regions restricted further analysis of these QTLs. For example, thirteen genes including 8 of unknown function were identified in the region on SSC1. Only a few (1 - 3) genes were detected in the other regions and none of them harbours SNPs on the 60K SNP panel. Previous GWAS studies in the pig have reported a few QTLs besides SSC15 to affect meat quality in different pig breeds/populations. Nonneman et al. [16] detected regions on SSC1 and SSC6 associated with multiple meat quality traits in a Landrace-Duroc-Yorkshire population. Sanchez et al. [17] identified seven QTLs on SSC1, SSC4, SSC8, SSC9 and SSC13 affecting meat pH and colour in French Large White (n = 385). Other chromosome regions affecting meat pH and/or colour include SSC3 [15], SSC2 and SSC6 [18], SSC4 in Swiss Large White Boars (n = 192) [14] and SSC6 in Pietrain boars [22]. These results indicate that meat pH and colour are determined by multiple genomic regions with relatively small effects, and that the genetic variants might be different in different pig breeds or populations. This complexity should be taken into account when designing a marker assisted selection program for specific breeds or populations.

### Further analysis of the major QTL on SSC15

Further analysis of the SSC15 region showed that three SNPs (20, 21 and 27) in very strong LD captured nearly all of the genetic variance of this region. SNP21 (MARC0083357), one of the closest SNPs to *PRKAG3* in the 60K panel, showed significant effects on all of the traits studied. *PRKAG3* was previously reported as a candidate gene affecting meat quality. The most important substitution (R200Q) in the gene caused a 70% increase in muscle glycogen in RN^−^ homozygous and heterozygous pigs that resulted in lower ultimate pH and water-holding capacity [4]. Another three mutations (T30N, G52S, I199V) in *PRKAG3* also showed significant effects on pH, and Minolta L* and b* for both loin and ham [1,5]. The most recent study [23] on *PRKAG3* detected several novel mutations by sequencing the coding and promoter regions, and the haplotype (*g.-157C* - *g.-58A* - *24E* - *199I*) was found to have a positive effect on meat quality in pigs. In our study, the most significant effects of SSC15_133 region reported here are on a*, b* and pH, but not lightness (L*) (Table 1). However, we also found evidence for effects of this region (SNP21) on other pork quality traits with P < 0.01 (see Additional file 1: Table S2), such as cooking loss, drip loss, shear force, colour a* for both fresh and thawed loin, colour b* on fresh loin, and colour (L*, a*, b*) on ham *gluteus medius*.

The most likely hypothesis is that the main effect of the QTL detected on SSC15 is due to the effect of polymorphism in *PRKAG3*. The three SNPs (SNPs 20, 21 and 27) might be in high LD with some of the mutations in *PRKAG3* that affect pH and colour. SNP20 (ASGA0070625), in complete LD with SNP21 in this study, was previously reported to affect meat pH in Yorkshire (n = 220) and Finnish Landrace (n = 230) [18]. However, further analysis [23] showed that ASGA0070625 was in very weak LD with the mutations of T30N, G53S and I199V in *PRKAG3*, but was in complete LD with some novel mutations such as g.-157C > G, g.-58A > G and K24E. These results indicate that there may be other polymorphisms and/or genes associated with variation in meat quality traits. Additional genes in this region may contribute to variation of meat quality independently or by interacting with *PRKAG3*. Three other known genes (*TTLL7, STK36* and *ZNF142*) are much closer to SNP21 than *PRKAG3*, and furthermore, SNP20 (ASGA0070625) is located in the intron 2 of *ZNF142* (zinc finger protein 142). *ZNF142* was reported as a putative candidate gene for both developmental and malignant disorders in human [24]. ZNF is one of the most abundant classes of transcriptional factors that regulate cell growth and differentiation, and *ZNF142* is involved in calcium ion binding and cell membrane integrity. The *STK36* (Serine/Threonine Kinase 36) gene product is related to the hedgehog signaling pathway and activation of cAMP-dependent PKA. *TTLL7* is Member 7 of the Tubulin Tyrosine Ligase-Like Family. SNP27 (MARC0039273) is close to *CDK5R2* (Cyclin-Dependent Kinase 5, Regulatory Subunit 2 (P39)), a neuron-specific activator of CDK5 kinase which is associated with cyclin-dependent kinase activating proteins. All these functions are associated with signal transduction, energy supply or cellular maintenance, which may play important roles on the activity of muscle cells post-mortem.

When SNP21 was fitted in the model, another 6 SNPs in this region were still significant for at least one of the studied traits. When the phenotypic LSMs were compared among the different haplotypes constructed with SNP21 and these SNPs, the differences increased compared with the difference among the three genotypes of SNP21. These results indicate that the other SNPs in this region contributed some effect on the traits, and these effects might be due to other genes in this region. Four of the six SNPs are intragenic with *ARPC2, TMBIM1, NRAMP1* and *VIL1*, and two are close to *RUFY4* and *CDK5R2. ARPC2* (actin related protein 2/3 complex, subunit 2) encodes one of seven subunits of the human Arp2/3 protein complex, which has been implicated in the control of actin polymerization in cells. Actin polymerization and actin/myosin interaction are strongly related to the toughening of muscle during the first 24 h post-mortem [25], consequently affecting meat pH and colour. The orthologue of *NRAMP1* (natural resistance-associated macrophage protein one) in human and other species is *SLC11A1*, which is a member of the solute carrier family 11 and encodes a multi-pass membrane protein that regulates intracellular pathogen proliferation and macrophage inflammatory responses [26]. *VIL1* (Villin 1) encodes a member of a family of calcium-regulated actin-binding proteins, it is a major actin-modifying protein associated with the microvillar actin filaments, and regulates epithelial cell morphology, actin reorganization, and cell motility through the functions in the capping, severing, and bundling of actin filaments [27]. These activities are very important to regulate cell death and enhance motility of actin-based proteins to maintain cellular structure and functionality. *RUFY4* (RUN and FYVE domain containing 4) is affiliated with the lncRNA class of proteins with roles in metal ion binding. Therefore, the main functions of these genes are related to membrane components and functionality, transporter activities and calcium ion binding, which play important roles in muscle activity related to structural integrity. These may therefore be involved in the dramatic micro-environmental changes that occur post-mortem [28].

### Gene network for this region of SSC15

The gene network constructed for the genes identified in the region on SSC15 showed strong interactions among them. *PRKAG3* is one of the major hubs in the network connecting with several of the genes found in this region of SSC15 such as *TTLL7, VIL1,* and *SLC11A1.* The protein encoded by *PRKAG3* is a regulatory subunit of adenosine monophosphate kinase (AMPK) which is an important energy-sensing enzyme that monitors cellular energy status and responses to cellular metabolic stresses. Activated AMPK is expected to inhibit glycogen synthesis and stimulate glycogen degradation. Studies in pig suggested that *PRKAG3* may play a key role in the regulation of energy metabolism in skeletal muscle [29], which is directly associated with the shift of the glycolysis between aerobic and anaerobic after exsanguination and consequently impact meat pH, colour and water holding capability [28]. Other genes in the network are directly or indirectly associated with a second major hub, *UBC* (ubiquitin C)*.* The important biological processes associated with *UBC* include activation of mitogen-activated protein kinase, cellular response to hypoxia, energy homeostasis and ion transmembrane transport, which are strongly associated with the muscle cell activities that are likely to be impacted post-mortem. These two major hubs are linked by *APP* (amyloid beta (A4) precursor protein) through AMPK subunits (e.g. *PRKAB1* and *PRKAB2)* and kinesin (e.g. *KLC1* and *KLC2*), also through some mediators associated with energy homeostasis like Ca^2+^ and ATP. These findings indicate that the genes identified in this region of SSC15 have strong interactions with *PRKAG3* and *UBC*. Based on their annotations, they may work together to impact meat quality through processes related to the regulation of actin filament polymerization, protein/ion/ATP binding, kinase activities and maintaining the cytoskeleton (Additional file 1: Table S1). All these processes are essential to maintain muscle membrane and myofibrillar protein integrity in the face of dramatic metabolic changes after exsanguination, such as the shift from aerobic to anaerobic glycolysis with anoxia and high body temperature, depletion of ATP, accumulation of lactate and hydrogen ions, and the consequent lowering of pH and lightening of meat colour [28,30].

## Conclusions

The most significant genomic region affecting both fresh and thawed loin pH, colour a* and b* for fresh ham and colour b* measured on thawed loin muscle has been identified on SSC15 spanning 133 - 134 Mb. Three SNPs (ASGA0070625, MARC0083357 and MARC0039273) with almost complete LD were the markers most strongly associated with the effect in this region and could be considered for marker assisted selection for meat quality improvement. An additional six SNPs also showed significant effects. Ten known genes have been identified to contain or be close to these markers, including the previously reported candidate gene *PRKAG3* along with *ZNF142, TMBIM1, NRAMP1, VIL1* and *ARPC2*. These genes appear to be networked through super-pathways of cell-to-cell signaling and interaction, cellular function and maintenance. They may play important roles in maintaining the integral architecture and functionality of muscle cells during the dramatic changes in the microenvironment post-mortem. These findings significantly contribute to our knowledge of the genomic architecture of this region and will potentially lead to new insights of the molecular mechanisms regulating meat pH and colour. Re-sequencing of this region may help resolve the molecular architecture of the region and identify the causative mutations.

## Methods

### Animals and phenotypic data

All animal procedures related to this project were reviewed and approved by the Canada Animal Care and Use Committee.

A total of 1943 crossbred commercial pigs from two populations (961 samples from Hypor and 982 samples from Genesus) were obtained for this study. All animals were from a typical Canadian three-way cross consisting of a Duroc sire line on an F1 hybrid female (Landrace * Large White). Pigs in both populations were fed *ad libitum* and harvested from 2010 to 2012. All animals were sent to the same processing plant when the body weight was close to 115 kg. Carcasses were processed within 24 h post-mortem and fresh meat measurements taken at that time, and pork loins (4^th^ - 7^th^ ribs) were harvested from each pig, vacuum packaged, and chilled (2 - 4°C) within 24 hours after exsanguination. All the packaged loins in the same batch were simultaneously frozen (−20°C) within 24 hours after exsanguination and maintained frozen until meat quality measurement. Prior to testing, the pork loins were thawed for 72 hours at 4°C. All samples were treated in the same way.

Fresh meat pH 24 h post-mortem (FpH24) was measured at two different locations on the surface of the loin muscle at the 10th rib. The average was used for the final statistical analysis. Minolta colours L*, a* and b* were measured at 24 h after exsanguination on the *longissimus dorsi* muscle (FMCOL) and subcutaneous fat above the *longissimus dorsi* muscle (FCOL) on the fresh loin, and on the muscle of *gluteus medius* (GMCOL), *quadriceps femoris* (QFCOL) and *iliopsoas* (ICOL) on the fresh ham face. Thawed muscle pH (TMpH) and colours (TMCOL L*, a*, b*) were measured on the thawed loin muscle. pH was measured using a portable pH meter (Fisher Scientific Company, Toronto, Ontario) equipped with a glass electrode (Hanna HI 98121, Hanna instruments, Canada) calibrated at room temperature using standards of pH 4.01 and 7.01. The pH probe was inserted about 5 cm into the centre of the loin three times through a small slit in three different locations within 2.5 cm from the posterior of the *longissimus* loin. The average was used as the final value for the analysis. Minolta colours were taken from three different locations on the surface of the muscle, and the average value was used for the final analysis. All the colour assessment was made using the colour system values specified by the Commission Internationale de L’eclairage (CIE) L* (lightness), a* (redness) and b* (yellowness), using a Konica Minolta Chroma-meter CR-400 (Konica Minolta Sensing Inc., Japan). Other traits including drip loss on both fresh (FMDL) and thawed (TMDL) loin muscle, cooking loss (CL) and shear force (SF) on thawed muscle were also measured in this project. The details of the measurement were described previously [31].

### Population stratification and LD estimation

Population stratification was analyzed using an identical-by-state (IBS) distance clustering method using the PLINK program [32]. Linkage disequilibrium (LD) of the pairwise SNPs was measured using r^2^ by *proc allele* program in SAS 9.3. The haplotype block pattern was built using haploView software [33].

### SNP array genotyping and filtering

Genomic DNA was isolated from tissue samples following the DNA Extraction instruction manual (Thermo Fisher Scientific Ltd., Ottawa, ON, Canada). Genotyping was conducted by Delta Genomics (Edmonton, AB, Canada) using Illumina PorcineSNP60 V2 Genotyping Beadchip according to the Illumina Infinium Assay (Illumina, Inc., San Diego, CA, USA).

Quality control for genotypes was performed with the criteria of genotyping call rate ≥ 95%, Chi-square of Hardy-Weinberg equilibrium test < 600, and minor allele frequency (MAF) ≥ 5%. After filtering, 1943 individuals and 42456 SNPs remained in the model for the final analysis.

SNP positions were updated according to the newest release from Ensembl (Sscrofa 10.2 genome version). Map information was adjusted based on the LD decay for each chromosome. Only the SNPs with reliable positions were used in this study. The missing genotypes were imputed by Fimpute v2.2 [34].

### Quality control

Traits of TMpH and FMDL did not exhibit normal distribution and were transformed using cosine and natural logarithm functions, respectively. All the records were adjusted within population using GLM procedure of SAS 9.3. A contemporary group consisting of slaughter batch, year and group during the test (~70 – 115 kg) was treated as a fixed effect in the model. There were 57 contemporary groups for Genesus and 46 for Hypor. Fixed effects of sex and contemporary group were fitted in the model below. For the Hypor population, the room and pen are fitted in the model as well, as they are available and have significant effect on the traits. The interactions among these factors were not significant and ignored in the model.

$$ {y}_{ij\;k\;l}=\mu +{s}_i+{\mathit{\mathsf{g}}}_j+{r}_k+{p}_l+{e}_{ij\;k\;l} $$

where *y*_*ijkl*_ is phenotypic observation, *μ* is overall mean, *s*_*i*_ is the effect of the *i*^*th*^ sex (1 = male and 2 = female), *g*_*j*_ is the effect of the *j*^*th*^ contemporary group (j = 1 - 57 for Genesus and 58 - 103 for Hypor), *r*_*k*_ is effect of the *k*^*th*^ room (k =1 - 10 for Hypor), *p*_*l*_ is the effect of the *l*^*th*^ pen (l = 1 - 13 for Hypor), and *e*_*ijkl*_ is the random residual error $\Big[{e}_{ijkl} \sim N\left(0,\ {\sigma}_e^2\Big)\right]$. The adjusted phenotype calculated as residual plus mean was used for the subsequent association studies.

### Genome-wide association analyses

The adjusted phenotype for each population were combined together and considered as a new phenotype for the genome-wide association. Population (Hypor and Genesus) was fitted as a fixed effect in the model. The marker effects were estimated by fitting all SNPs simultaneously using Bayesian methods as follow, as implemented in the online software GenSel.

$$ y=Xb+{\displaystyle \sum_j^k{z}_j{\alpha}_j{\delta}_j+\varepsilon } $$

where, ***y*** = vector of adjusted phenotypic observations, ***X*** = incidence matrix relating fixed factors to phenotypes, ***b*** = vector of fixed effects (mean and population), ***z***_***j***_ = vector of the genotype covariate for SNP *j* (*j* = 1 to k) based on the number of B alleles using Illumina’s (San Diego, California) genotype calling (coded 0, 1, 2), ***α***_***j***_ = allele substitution effect for SNP *j*, and ***δ***_***j***_ = indicator for whether SNP *j* was included (***δ***_***j***_ = 1) or excluded (***δ***_***j***_ = 0) in the model for a given iteration of the Markov chain Monte Carlo (MCMC). A total of 50 000 iterations were run for each analysis, with the first 5000 iterations discarded as burn-in. The probability of ***δ***_***i***_ = 0 was set equal to π = 0.99. The Bayesian model was implemented using method Bayes B. Genomic regions associated with traits were identified using 1 Mb, non-overlapping windows using Ensembl build 10.2 of the swine genome.

### Further marker effect and haplotype analysis

Further analysis on the SNPs within a 1 Mb window was implemented by a mixed animal model in ASREML [35]. The model equation was: *Y = Xb + Za + e*, where *Y* is the vector of adjusted phenotypes, *b* is the vector of fixed effects including mean, population and fitted SNPs, *a* is the random polygenic effects with $\Big[u \sim N\left(0,\ \boldsymbol{G}{\sigma}_{\mathrm{a}}^2\right]$, where ***G*** is the relationship matrix that was constructed based on pedigree information, and ${\sigma}_a^2$ is the polygenetic additive variance, *e* is the vector of residual errors with a distribution of $\Big[e\sim N\left(0,\ I{\sigma}_e^2\Big)\right]$, where ***I*** is the identity matrix and ${\sigma}_e^2$ is the residual variance. **X** and **Z** are the incidence matrices associated with *b* and *a*.

Window genomic estimated breeding value (GEBV) was calculated by sum of the marker effects of the SNPs within the window. And then the window GEBV was regressed on each SNP variant (0/1/2) within that window. The R-squared (RSQ) for the regression model, defined as the percentage of the GEBV variance explained by the SNP, was used to detect the most likely SNP in that window. The marker effect for the most likely SNP was estimated by fitting the SNP as fixed factor in the above animal model in ASREML. Then each of the remaining SNP was included one at a time in the model to test their significance after accounting for the effect of the most likely SNP. Finally, all the significant SNPs were simultaneously fitted in the model to retest their significance for the traits. Only the significant SNPs from the multiple marker association were used to construct the haplotypes. The least squares mean (LSMs) of the phenotype for each haplotype were estimated and contrasted by GLM procedure in SAS9.3. The significant threshold was set at P < 0.05.

### Post-GWAS bioinformatics analysis

Candidate gene identification and functional annotation for the significant SNPs were obtained using Ensembl annotation of Sscrofa 10.2 genome version (http://www.ensembl.org/biomart/martview). Gene networks were explored by the online software Ingenuity Pathways Analysis (IPA) (http://www.ingenuity.com/products/ipa). Human genes were used as background in pathway and gene network investigation, because some genes have not been characterized in pigs and translational gene aspects are of high interest. The functions and related information about candidate genes were summarized using the database of GENECARDS the human gene compendium (http://www.genecards.org/).

### Availability of supporting data

The original data sets supporting the results of this article are available. They are not public, but are able to be accessed by request for result verification only.

## Additional file

Additional file 1: Table S1. Functional annotation of the candidate and/or nearest genes to the significant SNPs in the region on SSC15. **Table S2.** Significant effects of SNP21 (MARC0083357) on other meat quality traits.

## Abbreviations

GWAS: Genome-wide association studies
LD: Linkage disequilibrium
IPA: Ingenuity pathways analysis
LSMs: Least squares mean
SNP: Single nucleotide polymorphism
SSC: *Sus scrofa* chromosome
QTL: Quantitative trait loci
IBS: Identity-by-State
GEBV: Genomic estimated breeding value
GO: Gene ontology
RSQ: R-squared
