# Supplementary material for: Genome-wide association studies (GWAS) identify a QTL close to PRKAG3 affecting meat pH and colour in crossbred commercial pigs
Source: BMC Genet. 2015 Apr 7;16:33. doi: 10.1186/s12863-015-0192-1 (PMC4393631; doi:10.1186/s12863-015-0192-1)
Supplement: Additional file 1: Table S1. — Functional annotation of the candidate and/or nearest genes to the significant SNPs in the region on SSC15. Table S2. Significant effects of SNP21 (MARC0083357) on other meat quality traits. [file 12863_2015_192_MOESM1_ESM.doc]

Additional file 1

Table S1 Functional annotation of the candidate and/or nearest genes to the significant SNPs in the region on SSC15.

| ***Gene*** | **GO Term Accession** | **GO Term Name** | **GO category** |
| --- | --- | --- | --- |
| *RUFY4* | GO:0046872 | metal ion binding | GOTERM_MF_FAT |
| *ARPC2* | GO:0030833 | regulation of actin filament polymerization | GOTERM_MF_FAT |
|  | GO:1900026 | positive regulation of substrate adhesion-dependent cell spreading | GOTERM_MF_FAT |
|  | GO:0005737 | cytoplasm | GOTERM_CC_FAT |
|  | GO:0005768 | endosome | GOTERM_CC_FAT |
|  | GO:0005856 | cytoskeleton | GOTERM_CC_FAT |
|  | GO:0005886 | plasma membrane | GOTERM_CC_FAT |
|  | GO:0005925 | focal adhesion | GOTERM_CC_FAT |
|  | GO:0031252 | cell leading edge | GOTERM_CC_FAT |
|  | GO:0005515 | protein binding | GOTERM_BP_FAT |
| *NRAMP1* | GO:0016020 | membrane | GOTERM_CC_FAT |
|  | GO:0005215 | transporter activity | GOTERM_MF_FAT |
| *VIL1* | GO:0009617 | response to bacterium | GOTERM_BP_FAT |
|  | GO:0061041 | regulation of wound healing | GOTERM_BP_FAT |
|  | GO:2000392 | regulation of lamellipodium morphogenesis | GOTERM_BP_FAT |
|  | GO:0008360 | regulation of cell shape | GOTERM_BP_FAT |
|  | GO:0051125 | regulation of actin nucleation | GOTERM_BP_FAT |
|  | GO:0010634 | positive regulation of epithelial cell migration | GOTERM_BP_FAT |
|  | GO:0030335 | positive regulation of cell migration | GOTERM_BP_FAT |
|  | GO:0032233 | positive regulation of actin filament bundle assembly | GOTERM_BP_FAT |
|  | GO:0043154 | negative regulation of cysteine-type endopeptidase activity involved in apoptotic process | GOTERM_BP_FAT |
|  | GO:0007173 | epidermal growth factor receptor signaling pathway | GOTERM_BP_FAT |
|  | GO:0007010 | cytoskeleton organization | GOTERM_BP_FAT |
|  | GO:0060327 | cytoplasmic actin-based contraction involved in cell motility | GOTERM_BP_FAT |
|  | GO:0035729 | cellular response to hepatocyte growth factor stimulus | GOTERM_BP_FAT |
|  | GO:0071364 | cellular response to epidermal growth factor stimulus | GOTERM_BP_FAT |
|  | GO:0051014 | actin filament severing | GOTERM_BP_FAT |
|  | GO:0030041 | actin filament polymerization | GOTERM_BP_FAT |
|  | GO:0030042 | actin filament depolymerization | GOTERM_BP_FAT |
|  | GO:0051693 | actin filament capping | GOTERM_BP_FAT |
|  | GO:0001726 | ruffle | GOTERM_CC_FAT |
|  | GO:0005902 | microvillus | GOTERM_CC_FAT |
|  | GO:0030027 | lamellipodium | GOTERM_CC_FAT |
|  | GO:0030175 | filopodium | GOTERM_CC_FAT |
|  | GO:0032432 | actin filament bundle | GOTERM_CC_FAT |
|  | GO:0032433 | filopodium tip | GOTERM_CC_FAT |
|  | GO:0003779 | actin and cytoskeletal protein binding | GOTERM_MF_FAT |
|  | GO:0005509 | calcium ion binding | GOTERM_MF_FAT |
|  | GO:0005515 | protein binding | GOTERM_MF_FAT |
|  | GO:0005546 | phosphatidylinositol-4,5-bisphosphate binding | GOTERM_MF_FAT |
|  | GO:0035727 | lysophosphatidic acid binding | GOTERM_MF_FAT |
|  | GO:0042802 | identical protein binding | GOTERM_MF_FAT |
|  | GO:0042803 | protein homodimerization activity | GOTERM_MF_FAT |
|  | GO:0043027 | cysteine-type endopeptidase inhibitor activity involved in apoptotic process | GOTERM_MF_FAT |
|  | GO:0051015 | actin filament binding, cytoskeletal protein binding | GOTERM_MF_FAT |
| *ZNF142* | [GO:0008270](http://amigo.geneontology.org/cgi-bin/amigo/term_details?term=GO:0008270) | zinc ion binding | GOTERM_MF_FAT |
|  | [GO:0046872](http://amigo.geneontology.org/cgi-bin/amigo/term_details?term=GO:0046872) | metal ion binding | GOTERM_MF_FAT |
| *STK36* | GO:0006468 | protein phosphorylation | GOTERM_BP_FAT |
|  | GO:0004672 | protein kinase activity | GOTERM_MF_FAT |
|  | GO:0004713 | protein tyrosine kinase activity | GOTERM_MF_FAT |
|  | GO:0005524 | ATP binding, ion binding | GOTERM_MF_FAT |
|  | GO:0016772 | transferase activity, transferring phosphorus-containing groups | GOTERM_MF_FAT |
| *TTLL7* | GO:0018095 | protein polyglutamylation | GOTERM_BP_FAT |
|  | GO:0006464 | cellular protein modification process | GOTERM_BP_FAT |
| *PRKAG3* | GO:0030554 | adenyl nucleotide binding | GOTERM_MF_FAT |
| *CDK5R2* | GO:0045860 | positive regulation of protein kinase activity | GOTERM_BP_FAT |
|  | GO:0045956 | positive regulation of calcium ion-dependent exocytosis | GOTERM_BP_FAT |
|  | GO:0005737 | cytoplasm | GOTERM_CC_FAT |
|  | GO:0016533 | cyclin-dependent protein kinase 5 holoenzyme complex | GOTERM_CC_FAT |
|  | GO:0016534 | cyclin-dependent protein kinase 5 activator activity | GOTERM_MF_FAT |

Table S2 Significant effects of SNP21 (**MARC0083357**) on other meat quality traits.

| **Traits** | **Full name** | **AA (n = 476)** | **AB (n = 958)** | **BB (n = 457)** | **Additive** | **%_PheVar** |
| --- | --- | --- | --- | --- | --- | --- |
| TMDL | Drip loss on thawed loin muscle | 5.698±0.101**^a^** | 5.948±0.071**^ab^** | 6.153±0.098**^b^** | -0.228±0.070**^*^** | 0.52 |
| FMDL ^#1^ | Drip loss on fresh loin muscle | 0.188±0.015**^A^** | 0.226±0.010**^AB^** | 0.274±0.014**^B^** | -0.043±0.010**^**^** | 0.73 |
| CL | Cooking loss on thawed loin muscle | 26.968±0.105**^a^** | 27.182±0.074**^ab^** | 27.451±0.102**^b^** | -0.241±0.073**^**^** | 0.53 |
| SF | Shear force on thawed loin muscle | 45.401±0.429**^a^** | 46.381±0.303**^ab^** | 47.213±0.416**^b^** | -0.906±0.298**^*^** | 0.42 |
| TMCOL a* | Thawed loin muscle Minolta colour a* | 7.775±0.043**^A^** | 7.874±0.030**^AB^** | 8.002±0.041**^B^** | -0.113±0.030**^**^** | 0.70 |
| FMCOL a* | Fresh loin muscle Minolta colour a* | 7.378±0.054**^A^** | 7.633±0.038**^B^** | 7.727±0.052**^B^** | -0.175±0.038**^**^** | 1.10 |
| FMCOL b* | Fresh loin muscle Minolta colour b* | 17.152±0.053**^a^** | 17.343±0.038**^b^** | 17.344±0.052**^ab^** | -0.096±0.037**^*^** | 0.41 |
| GMCOL L* | *gluteus medius* Minolta colour L* on fresh ham | 44.625±0.098**^A^** | 44.835±0.069**^A^** | 45.230±0.095**^B^** | -0.302±0.068**^**^** | 1.02 |
| GMCOL a* | *gluteus medius* Minolta colour a* on fresh ham | 6.903±0.050**^A^** | 7.065±0.035**^AB^** | 7.215±0.048**^B^** | -0.156±0.035**^**^** | 0.76 |
| GMCOL b* | *gluteus medius* Minolta colour b* on fresh ham | 15.043±0.041**^A^** | 15.149±0.029**^A^** | 15.343±0.040**^B^** | -0.150±0.029**^**^** | 1.29 |
| QFCOL L* | *quadriceps femoris* Minolta colour L* on fresh ham | 48.421±0.138**^a^** | 48.945±0.097**^b^** | 49.009±0.133**^b^** | -0.294±0.096**^**^** | 0.46 |

^#1^: phenotype was adjusted by natural logarithm function to meet the normal distribution.

different subscript capital letters (A, B) among three genotypes in each row means the difference at the significant level of *P < 0.001*; different subscript small letters among three genotypes means the difference at the significant level of *P < 0.01*.

For additive effect, ** means significant level of *P < 0.001*; * means significant level of *P < 0.01*.
